# Supplementary material for: Discrepancy in Sterol Usage between Two Polyphagous Caterpillars, Mythimna separata and Spodoptera frugiperda
Source: Insects. 2022 Sep 27;13(10):876. doi: 10.3390/insects13100876 (PMC9604351; doi:10.3390/insects13100876)
Supplement: Supplementary file 1 [file insects-13-00876-s001.zip › insects-1892363-supplementary.pdf]

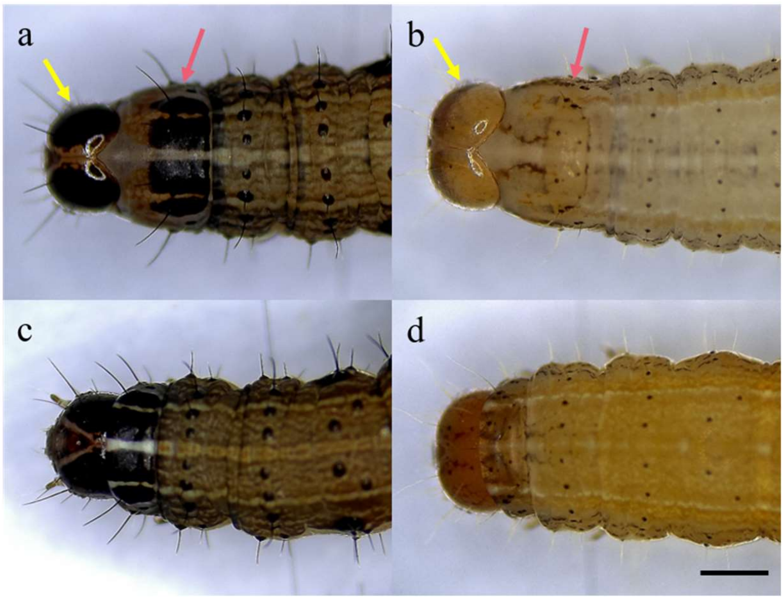

Figure S1. Morphological character for identifying the molting stage. The larva, which is ready to molt to the 4th instar, stops feeding and evacuates its intestine. It forms a new head capsule (pointed by red arrows), and pushes the old head capsule (pointed by yellow arrows) anteriorly ((a) *Spodoptera frugiperda*; (b) *Mythimna separata*). The head capsule of the larva in the middle stage of the 3rd instar does not have this morphology ((c) *S. frugiperda*; (d) *M. separata*). Scale bar, 1 mm.

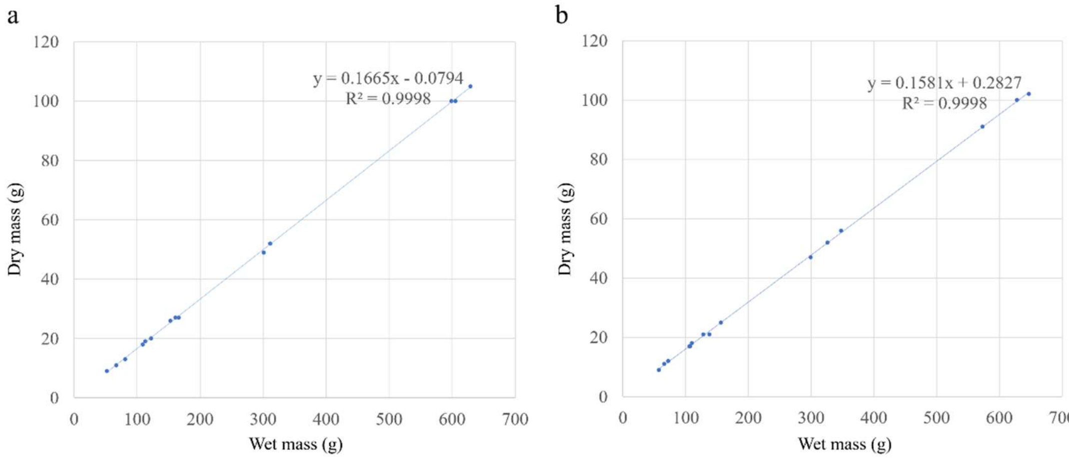

Figure S2. The regression line between the wet and dry mass of cholestanone (a) and cholesterol (b) diets.

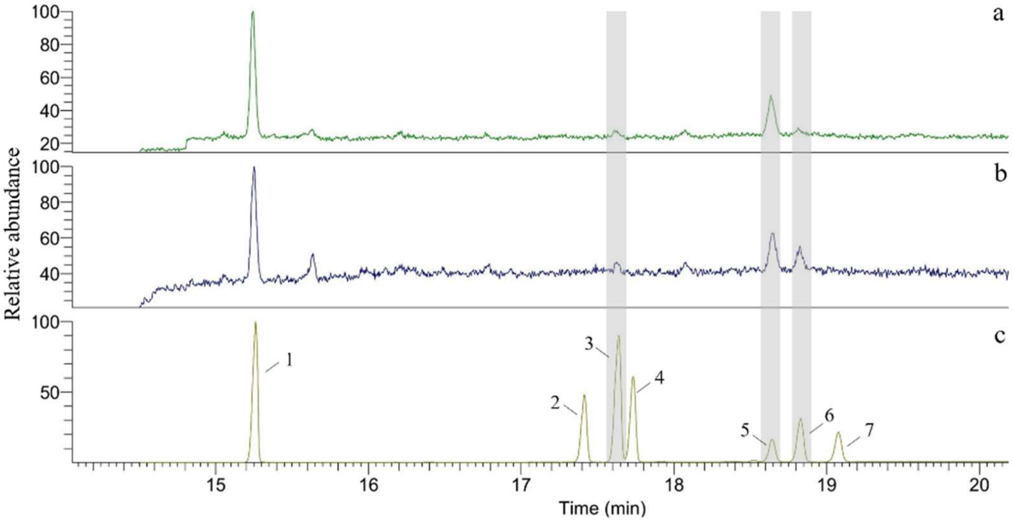

Figure S3. GC/MS analysis of sterol/steroid profiles in *Mythimna separata* and *Spodoptera frugiperda*. Body sterol/steroid profile of *M. separata* (a) and *S. frugiperda* (b) on cholestanone diet. (c) Chromatograms of the position of the standards used in this study including (1) cholestane; (2) 5β-cholestan-3β-ol; (3) 5α-cholestan-3α-ol (epi-cholestanol); (4) 5β-cholestan-3α-ol; (5) cholesterol; (6) 5α-cholestan-3β-ol (cholestanol); (7) cholestanone. Cholestane (10 μg) as the internal standard was added into each sample to standardize sterols/steroids.

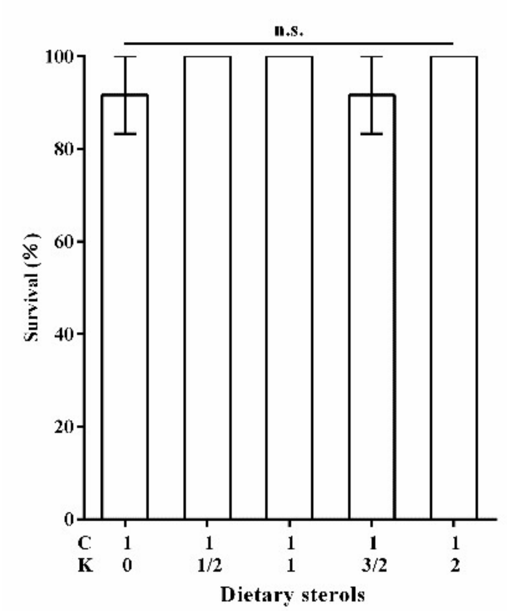

Figure S4. *Mythimna separata* larval survival on multiple mixed diets containing cholesterol and cholestanone. Upon hatching, *M. separata* larvae were fed different diets, and their survival was recorded on the 12th day. n.s. means no significant difference at $p < 0.05$.
